# A Multicomponent, Multi-Trigger Intervention to Enhance Asthma Control in High-Risk African American Children

**DOI:** 10.5888/pcd16.180387

**Published:** 2019-05-30

**Authors:** Mark H. Ebell, Stephanie Patrice Hall, R. Chris Rustin, Kia Powell-Threets, Luis Munoz, Kia Toodle, (Mary) Lu Meng, Jean O’Connor

**Affiliations:** 1 College of Public Health, University of Georgia, Athens, Georgia; 2 Chronic Disease Prevention Section, Georgia Department of Public Health, Atlanta, Georgia; 3 Environmental Health Services Branch, Georgia Department of Public Health, Atlanta, Georgia

## Abstract

**Introduction:**

We evaluated the effectiveness and feasibility of implementation of a multicomponent, multi-trigger (MCMT) intervention through a public health department in a high risk population of African American children.

**Methods:**

This was a pragmatic quasi-experimental pretest–posttest study. The population consisted of African American children enrolled in Medicaid and Children’s Medical Services who had poorly controlled asthma. The MCMT intervention included 4 educational sessions and home asthma trigger reduction. Parents reported outcomes at baseline and at 1 to 3 months, 6 months, and 12 months after the MCMT intervention. Analysis used the McNemar χ^2^ test and Student *t* test for paired observations. Data were collected during 2014 through 2016 in Augusta, Georgia.

**Results:**

The number of children with asthma that was assessed as well controlled increased from 4 to 17 out of 20 (*P* < .001). Compared with baseline, at 12 months parents reported fewer days of school missed (6.4 vs 4.2, *P* = .01), fewer emergency department visits (1.7 vs 0.6, *P* = .02) and fewer hospitalizations (0.59 vs 0.18, *P* = .05). The most common environmental interventions were dust mitigation, getting a mattress or pillow protector, and cockroach mitigation.

**Conclusion:**

An MCMT intervention in high risk African American children with poorly controlled asthma administered through the health department was associated with significant improvements in asthma control, days of school missed, and emergency department visits. Broader implementation of these strategies is warranted.

**Summary**

**What is already known on this topic?**

For children with uncontrolled asthma, combining education with trigger reduction strategies (multicomponent multi-trigger [MCMT] interventions) has been effective.

**What is added by this report?**

We demonstrate the effectiveness and feasibility of a MCMT intervention offered through a public health department to African American children enrolled in Medicaid. Children receiving the intervention had fewer missed days of school, emergency department visits, and hospitalizations.

**What are the implications for public health practice?**

Our report demonstrates the value to Medicaid and state and local health departments in collaborating to offer these types of interventions for high-risk children with asthma.

## Introduction

Asthma is a common chronic condition characterized by bronchospasm and inflammation, typically accompanied by intermittent exacerbations. Asthma may impair quality of life and function, may result in hospitalization, and rarely can cause death. African American children have a 60% greater prevalence of asthma than non-Hispanic white children. They also have a 4.5-fold greater likelihood of hospital admission and a 7.1-fold greater likelihood of death attributable to asthma, with 9.2 deaths per million African American children per year. When limiting the population to children aged 14 years or younger, mortality was 10 times higher for African American children (1).

A group at especially high risk is children who live in poverty, who may have greater exposure to dust and other allergens in the home, and who may be less likely to have an asthma action plan, less likely to have a regular source of primary care, and less likely to have ready access to inhalers. Social determinants such as poverty and living situation adversely affect asthma-related outcomes (2–7).

Approximately 1 in 10 children in Georgia have asthma. In 2016, approximately half of these Georgia children with asthma were African American, and over 60% lived in households with an annual income less than $50,000. Also in 2016, nearly 18% lived in a household where at least 1 parent smoked. In 2015, the State of Georgia identified pediatric asthma as a public health priority, with focus on elimination of pediatric asthma mortality and reduction of repeat hospitalization and emergency department visit rates among its Medicaid population (8).

The Georgia Department of Public Health’s Chronic Disease Prevention Section sought a means to address the pediatric asthma priority and to improve asthma outcomes for high-risk children with poorly controlled asthma in an area known for very high risk of uncontrolled asthma and higher than expected pediatric asthma mortality, especially among African American children. The department undertook a pilot project to test the delivery of a multicomponent, multi-trigger (MCMT) intervention in an area of the state with a high burden of pediatric asthma. A systematic review concluded that MCMT interventions were effective in improving overall quality of life and productivity in children with asthma (9) and were cost-effective (10). These interventions were also recommended in 2008 by the Community Guide for Preventive Services for implementation (11). 

However, before this pilot project, MCMT interventions had not been implemented in the Medicaid population or by the public health system in Georgia. The purpose of this project was to assess the feasibility and outcomes that could be achieved through implementation of an MCMT intervention in a high-need, hard-to-reach population. MCMT interventions are directed at reaching, engaging, and educating children with poorly controlled asthma and their families, with the aim of reducing asthma-related emergency department visits and hospitalizations. This approach entails 1) identifying children with poorly controlled asthma, 2) linking them to health care providers who follow National Asthma Education and Prevention Program Expert Panel Report 3 guidelines–based care (12), 3) educating them on asthma self-management, 4) providing a supportive school environment, and 5) referring to or providing home trigger assessments and reduction services by environmental health specialists. We report the results of this pilot project.

## Methods

This was a pretest–posttest quasi-experimental trial. The Institutional Review Board of the Georgia Department of Public Health reviewed the study and approved it as exempt. Data were collected during 2014 through 2016 in Augusta, Georgia.

### Population and recruitment measures

Eligible children had English-speaking parents or guardians, were aged from 0 to 17 years, and resided in a high-burden health district in the state of Georgia. The district was selected on the basis of historical data regarding pediatric asthma mortality, pediatric asthma hospitalizations, and use of emergency departments for pediatric asthma. The district also had infrastructure available for implementation of the intervention. For inclusion in the study, children had to be dually enrolled in Children’s Medical Services (CMS), a case management program for children with special medical needs operated by the Georgia Department of Public Health for Medicaid, and Fee-for-Service Medicaid, and had to have diagnosed asthma that was either not well controlled or very poorly controlled. Not well controlled or poorly controlled was defined for the purposes of this project as having a hospitalization or multiple emergency department visits in the last 6 months, confirmed via an asthma control questionnaire that assessed symptoms more than 2 days a week; night time awakenings (one or more per month or week depending on age); interference with normal activity (some limitation); and an Asthma Control Test score of 19 or less (13). CMS was selected as the vehicle for enrollment because the program provides funding for certain durable medical supplies and other remediation materials, such as mattress and pillow covers, that might be needed to fully implement the intervention.

Children were identified by using existing lists of CMS enrollees who had their CMS Asthma Questionnaires on file, and their asthma status was ascertained by a public health nurse based on the existing CMS Asthma Questionnaires completed before the intervention. No children were excluded. The nurse then contacted the child’s parent or guardian to inform them of the opportunity to participate and to receive consent from interested families. On consenting to enroll in the program, the nurse then scheduled 4 education sessions on asthma self-management and 2 asthma healthy home assessments by environmental health specialists.

### Intervention and data collection

The MCMT intervention was 4 education sessions, using the Wee Breathers asthma curriculum (14), and 2 healthy home assessments (initial assessment and follow-up assessment). Education sessions were delivered in a group format from May through August 2016. At the first group session, parents and guardians signed the consent form and were given the Asthma Experience Questionnaire (15). The survey included questions about the child’s asthma (control, symptoms, quality of life for parents and guardians and children, number of emergency department visits, school and workdays missed because of asthma, asthma medications, school environment, and household information). They were then provided Lesson 1: Asthma Basics and Lesson 5: Asthma Action Plan from the Wee Breathers curriculum. Delivering Lesson 5 earlier is to make sure participants can set up their asthma action plans with their health care providers as soon as possible. The second session covered Lesson 2: Asthma Triggers and Lesson 3: Controlling Asthma Triggers from the Wee Breathers curriculum. The third session covered Lesson 4: Asthma Medicines and Lesson 6: Communication with the Asthma Team. In the last session (session 4), they were provided Lesson 7: Asthma Management Goals and a thorough review of the entire curriculum, and given the Asthma Experience Questionnaire and CMS asthma questionnaire again.

At each self-management education session, participants were assessed on proper use of medication devices and given pretests and posttests to assess knowledge they gained in each session. The pretests and posttests for the asthma self-management education sessions consisted of questions about the basics of asthma, triggers that make asthma worse, the importance of an asthma action plan, how to talk to the child’s health care team about asthma, and asthma medicines and devices. Children who assented participated in the education session along with their parents. After finishing the program, participants completed follow-up surveys at 1 to 3 months, 6 months, and 12 months by telephone or in person at the health department. Participants received a $25 gift card for completing the enrollment interview and a $50 gift card for completing the exit interview.

As part of the intervention, the Asthma Healthy Home Assessments were conducted to reduce asthma triggers at home. The assessments consisted of an initial assessment and a follow-up assessment conducted by environmental health specialists employed by the local health department in Augusta and trained by the Georgia Department of Public Health. In the initial assessment, the asthma triggers at home were documented by using a Healthy Homes assessment tool (16), and an In-Home Action Plan to improve the home environment was established with the parents or guardians. During the follow-up assessment, an environmental health specialist walked through the home to follow up on areas of concern identified during the first visit to determine if the recommendations were implemented and noted barriers to implementation.

On enrolling in the program, parents or guardians had the option of consenting to continued case management by the CMS nurse. If they agreed, the CMS nurse sent a letter to the child’s regular health care provider updating them on the child’s asthma and provided the child’s asthma action plan to the child’s school nurse. The nurse also helped to establish bidirectional communication with the child’s provider, reinforced self-management education lessons, and assessed guidelines-based care.

### Analysis

Numerous variables had a large amount of missing data; therefore, our analysis was limited to variables where most respondents provided data. Categorical data were dichotomized based on a review of the distribution of each variable, to increase statistical power given the small number of observations. The McNemar χ^2^ test for paired observations was used to test significance. Continuous data were analyzed by using the Student *t* test (one sided) for paired data, based on the hypothesis that there would be improvement in outcomes at follow-up. A *P* value of less than .05 was considered significant, and Stata version 14.0 (StataCorp LLC) was used for all analyses.

## Results

Of the 135 children screened, 46 were eligible to participate in the intervention. In all, 25 children were recruited for the study, and 23 completed the program, resulting in 23 participants with follow-up data obtained for all children. Of the 23 children, 14 were boys, 21 were African American, and 19 were non-Hispanic. Of 18 participants reporting their income, all had an estimated annual household income less than $30,000, and the head of household was described as the mother for 15 of 23 children. Only 4 households reported that they had smokers, and only 1 reported smoking inside the home.

There were clinically and statistically significant improvements in parental assessments of asthma control, frequency of nighttime awakenings, and activity limitation (Table 1). For example, the number of children whose asthma was assessed as being well controlled went from 4 out of 20 to 17 out of 20 (*P* < .001) (data were missing for 3 children).

**Table 1:** Children’s Medical Services Asthma Questionnaire Findings at Baseline and 6 and 12 Months’ Follow-up in Intervention to Enhance Asthma Control in High-Risk African American Children (n = 23), 2016

Outcome	Baseline	Follow-up	*P* Value
**Categorical variables, n/total^a^**			
Parental assessment that asthma is well controlled (vs not well or very poorly controlled)	4/20	17/20	<.001^b^
Nighttime awakenings once or fewer per month (vs more than once per month)	1/12	7/12	.014^b^
No activity limitations (vs some or severe limitations)	1/17	7/17	.014^b^
**Continuous variables, mean (95% confidence interval)**			
Days of school missed because of asthma in the last 6 months	3.3 (1.6–5.0)	1.4 (0.37–2.4)	.01^c^
Days of work missed by parent or caregiver because of child’s asthma in last 6 months	1.3 (0.10–2.5)	0.45 (0.0–2.3)	.12^c^
Emergency department visits in the past 6 months	0.95 (0.32–1.6)	0.27 (0.0–0.69)	.004^c^
Hospitalizations for asthma in the past 6 months	0.18 (0.01–0.36)	0.09 (0.0–0.28)	.16^c^
Days of school missed because of asthma in the last 12 months	6.4 (2.8–10.0)	4.2 (0.42–8.0)	.01^c^
Days of work missed by parent or caregiver because of child’s asthma in last 12 months	2.4 (0.48–4.2)	1.2 (0.0–2.5)	.08^c^
Emergency department visits in the past 12 months	1.7 (0.35–3.0)	0.64 (0.02–1.3)	.02^c^
Hospitalizations for asthma in the past 12 months	0.59 (0.05–1.1)	0.18 (0.0–0.56)	.05^c^

a Data were missing for some participants.

b McNemar χ^2^ test.

c Paired *t* test.

There were significant reductions in days of school missed (1.4 vs 3.3, *P* = .01) and emergency department visits in the past 6 months (0.27 vs 0.95, *P* = .004), with similar findings for emergency department visits in the past 12 months (Table 1). There were also fewer parent-reported hospitalizations for asthma in the past 12 months at the 12-month follow-up (0.18 vs 0.59, *P* = .05).

While the general trends regarding the frequency of daytime and nighttime symptoms was toward improvement in each of the 3 follow-up surveys, this difference was only significant for daytime symptoms at the 1 to 3 months follow-up (Table 2). Use of rescue inhalers decreased and use of controller inhalers increased, although none of these differences was significant because of the small sample size.

**Table 2:** Children’s Medical Services Asthma Questionnaire at Baseline and Follow-up in Intervention to Enhance Asthma Control in High-Risk African American Children, 2016

Outcome	Baseline	Follow-up	*P* Value^a^
**Categorical variables, n/total**			
**At 1 to 3 months follow-up**			
Daytime symptoms 2 times a week or less (vs more than 2 times a week)	14/23	21/23	.01
Nighttime symptoms 2 nights a month or less (vs more than 2 nights a month)	17/23	20/23	.26
**At 6 months follow-up**			
Daytime symptoms 2 times a week or less (vs more than 2 times a week)	14/23	15/23	.26
Nighttime symptoms 2 nights a month or less (vs more than 2 nights a month)	17/23	17/23	<.99
**At 12 months follow-up**			
Daytime symptoms 2 times a week or less (vs more than 2 times a week)	14/23	18/23	.21
Nighttime symptoms 2 nights a month or less (vs more than 2 nights a month)	17/23	19/23	.41
**Continuous variables, mean (95% confidence interval)**			
**At 1 to 3 months follow-up**			
Number of rescue inhaler uses in previous 14 days	4.2 (1.5–6.9)	3.5 (1.5–5.4)	.28
Number of controller inhaler uses in previous 14 days	11.4 (9.3–13.5)	12.6 (11.0–14.3)	.14
**At 6 months follow-up**			
Number of rescue inhaler uses in previous 14 days	4.4 (1.2–7.6)	4.1 (1.6–6.6)	.42
Number of controller inhaler uses in previous 14 days	11.2 (8.6–13.8)	12.3 (10.2–14.3)	.18
**At 12 months follow-up**			
Number of rescue inhaler uses in previous 14 days	4.4 (1.2–7.6)	3.0 (1.3–4.8)	.10
Number of controller inhaler uses in previous 14 days	11.2 (8.6–13.8)	12.2 (10.1–14.3)	.24

a McNemar χ^2^ test.

The most common action plan items that were recommendations from the healthy homes assessment were cleaning to mitigate dust and dirt (n = 14), getting a mattress or pillow protector (n = 10), and cockroach mitigation (n = 9) (Table 3). Of 69 recommendations, 37 had been fully mitigated and 7 were partially mitigated on the return visit.

**Table 3:** Action Plan Items Based on Environment and Client Response in Intervention to Enhance Asthma Control in High-Risk African American Children, 2016

Recommendation	No. of Times Recommended	Mitigation on Follow-up Visit			
		Yes	Partial	No	Not Reported or Lost to Follow-up
Cleaning to mitigate dust and dirt	14	10	2	1	1
Get a mattress and/or pillow protector	10	4	2	3	1
Cockroach mitigation	9	4	1	0	4
Get a vacuum cleaner with a high efficiency particulate air (HEPA) filter	6	2	0	4	0
Clean or remove plush toys	6	3	0	3	0
Fix water leaks	5	3	0	0	2
Other environmental measures^a^	5	1	1	2	1
Vent stove or dryer to outside	5	4	0	0	1
Furnace air filter replace or clean	4	1	1	2	0
Avoid toxic cleaning products	3	3	0	0	0
Asthma guidance provided	2	2	0	0	0
**Total**	**69**	**37**	**7**	**15**	**10**

a Included replacing window screens, removing pet from child’s room and bed, removing carpeting, removing scented candles, and smoking cessation.

## Discussion

This pilot project provided a MCMT intervention to 23 children who were dually enrolled in CMS and Medicaid in a single public health district and who had asthma that was either not well controlled or very poorly controlled. Compared with the baseline assessment, the follow-up assessment of the MCMT intervention found that parents reported clinically and statistically significant improvements in asthma control, frequency of nighttime awakenings, and activity limitations. In addition, they reported significantly fewer days of school missed (1.4 vs 3.3) and fewer emergency department visits (0.27 vs 0.95) at follow-up. While fewer hospitalizations were reported at 12 months follow-up, this finding was not significant. These findings are consistent with those in a previous systematic review (9), and are notable for having been implemented in a real world setting without research staff and in a very high-risk population.

The study had several limitations. First, it is a small and nonrepresentative sample of all children with asthma. However, we believe it does reflect a critical population that experiences a substantial burden of disease, namely African American children with poorly controlled asthma who live in poverty. Second, the reliance on parental self-report is another limitation and is subject to optimistic bias (parents may wish to please the interviewers by reporting positive results). Third, the absence of a control or comparison group is an important limitation. Finally, we had missing data, which is the result of doing a pragmatic study in a real-world setting executed by a staff without extensive research training.

To address these limitations, a larger trial is warranted, perhaps using a stepped-wedge design to compare results in treated and untreated homes. Additional study in the Medicaid managed care population is also needed, and a larger pilot project with 100 children is under way at the Georgia Department of Public Health. Longer term studies are also needed to evaluate the persistence of the intervention and whether changes in medication use and environmental improvements persist over time.

There is a gap between what is effective to control asthma and what is routine practice in community and clinical settings. MCMT interventions are multisectoral by definition, presenting challenges for implementation in real-world settings. Public health departments, while theoretically well positioned to lead the charge of bringing together clinical, environmental, housing, and educational supports with payors to promote the control of chronic conditions at the community level, often face the realities of limited resources, high staff turnover, limited leadership capacity, and hard-to-reach populations with severe health conditions. Our study team found that despite extremely limited resources, state and local health departments can collaborate together and with the Medicaid program to not only implement the program but to achieve what appear to be promising outcomes. In a state where pediatric asthma mortality is a considerable concern, and where outcomes for pediatric asthma are strongly divided along racial and economic lines (8), the modest success of this project should serve as an important lesson learned for other states, regardless of Medicaid expansion status. Furthermore, the project demonstrated that collaboration between nursing, chronic disease prevention, and environmental health professionals was feasible to jointly address pediatric asthma. However, considerable resources were expended on developing the methodology for the project to be applied in a real-world setting, and more practical guidance for state and local health departments on the implementation of MCMT interventions in real-world settings is needed. Adequate resources for further testing of widespread implementation of MCMT interventions for appropriate populations is needed, as is reimbursement for the interventions through Medicaid of the service delivery providers, including public health departments, if the efforts are to be sustainable.
